# Use of the Meganuclease I-SceI of *Saccharomyces* *cerevisiae* to select for gene deletions in actinomycetes

**DOI:** 10.1038/srep07100

**Published:** 2014-11-18

**Authors:** Lorena T. Fernández-Martínez, Mervyn J. Bibb

**Affiliations:** 1 Department of Molecular Microbiology, John Innes Centre, Norwich, NR4 7UH, U.K

## Abstract

The search for new natural products is leading to the isolation of novel actinomycete species, many of which will ultimately require genetic analysis. Some of these isolates will likely exhibit low intrinsic frequencies of homologous recombination and fail to sporulate under laboratory conditions, exacerbating the construction of targeted gene deletions and replacements in genetically uncharacterised strains. To facilitate the genetic manipulation of such species, we have developed an efficient method to generate gene or gene cluster deletions in actinomycetes by homologous recombination that does not introduce any other changes to the targeted organism's genome. We have synthesised a codon optimised I-SceI gene for expression in actinomycetes that results in the production of the yeast I-SceI homing endonuclease which produces double strand breaks at a unique introduced 18 base pair recognition sequence. Only those genomes that undergo homologous recombination survive, providing a powerful selection for recombinants, approximately half of which possess the desired mutant genotype. To demonstrate the efficacy and efficiency of the system, we deleted part of the gene cluster for the red-pigmented undecylprodiginine complex of compounds in *Streptomyces* *coelicolor* M1141. We believe that the system we have developed will be broadly applicable across a wide range of actinomycetes.

Actinomycetes are Gram-positive mycelial bacteria with a high genomic GC content that produce a wide variety of industrially and medically relevant compounds including chemotherapeutics, fungicides, herbicides, immunosuppressants and many clinically used antibiotics. The search for new pharmacologically active and industrially relevant natural products has led to the recent isolation of many novel actinomycete strains, many of which are recalcitrant to genetic manipulation.

Homologous recombination is a highly conserved process present in all forms of life, from bacteria to eukaryotes^1^. It is used regularly to target gene replacement and deletion in a wide range of organisms including bacteria^2^, although the efficiency of this process is very much dependent on the propensity of the targeted organism to carry out homologous recombination.

Gene deletion, replacement or disruption in bacteria generally involves the use of a non-replicative vector carrying the targeted DNA, with selection for antibiotic resistance resulting in integration of the construct into the host's genome by homologous recombination (yielding the first cross-over recombinant). The resulting strain is then screened for loss of the resistance marker carried on the vector, and the sensitive isolates obtained screened phenotypically and/or by PCR to identify the required second cross-over recombinant. In organisms with a low intrinsic level of homologous recombination, screening for loss of antibiotic resistance can be a very tedious process, and in mycelial actinomycetes that do not sporulate, sometimes extremely difficult if not impossible to achieve. To expedite this process and to select for this second recombination event, it is possible to induce a DNA double-strand break adjacent to a targeted locus by using a meganuclease such as I-SceI. I-SceI is a monomeric 235 amino acid homing endonuclease encoded in the mitochondrial DNA of *Saccharomyces* *cerevisiae*^3^ which is able to cut double stranded DNA at an 18 bp recognition sequence: TAGGGATAACAGGGTAAT. The length of this sequence ensures that it is absent from all actinomycete genome sequences determined thus far. Chromosome repair and viability can only result through homologous recombination between duplicated regions of the genome, thus providing a selection for the second cross-over event which should yield a mixture of both the original genotype and the required mutant.

Lu *et al*.^4^ previously reported the successful exploitation of the I-SceI meganuclease in *Streptomyces* *coelicolor* to delete the gene cluster for the blue-pigmented polyketide antibiotic actinorhodin using a two plasmid system in which the vector delivering the meganuclease, an integrative pSET derivative^5^, remained, with its antibiotic resistance gene, in the genome of the targeted organism once the required second cross-over recombinant had been obtained. This precludes the subsequent use in the mutant of any vector that relies on the widely-used integration system of the *Streptomyces* phage ΦC31, and eliminates the further use of the thiostrepton resistance marker carried by the pSET derivative. Moreover, integration at the ΦC31 *attB* site can considerably reduce the level of production of some specialised metabolites, including antibiotics^6^.

Here we report a I-SceI-based mutagenesis strategy that enables the construction of marker-less deletions of genes and gene clusters that is applicable to many actinomycete species, and that should be particularly useful for strains that are difficult to manipulate genetically.

## Results

### Validating the system with a marker-less deletion in *S. coelicolor*

**Figure 1:**
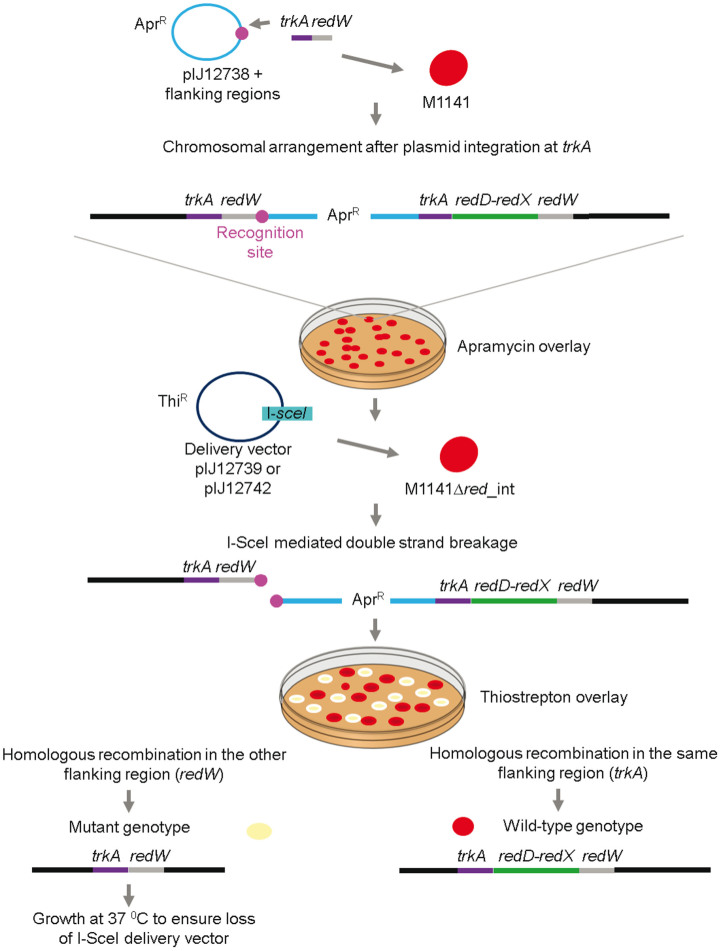
Overview of the protocol developed to obtain marker-less gene deletions in actinomycetes. pIJ12738 containing the I-SceI recognition sequence (shown by a pink circle) and the flanking regions (in this example, *trkA* and *redW*, corresponding to the left and right flanking regions of *redD* and *redX*, respectively) of the chromosomal region to be deleted was conjugated into M1141. Apramycin resistant exconjugants were analysed by PCR to confirm integration of the plasmid (in this schematic, integration occurred by homologous recombination at *trkA*). A single exconjugant (M1141Δ*red*_int) was selected as recipient of the delivery vectors pIJ12739 and pIJ12742 expressing the I-SceI meganuclease gene. After conjugation, the plate was overlaid with thiostrepton to select for exconjugants. I-SceI creates double strand breaks at its introduced recognition sequence, and the only genomes to survive are those that undergo homologous recombination to reconstitute an intact chromosome. If recombination occurs in the same flanking region that was used in the first cross-over event (in this example, *trkA*), then the exconjugant will revert to a wild type genotype. In contrast, if recombination takes place in the other flanking region (in our example, *redW*), the exconjugant will have a mutant genotype. Exconjugants were analysed by PCR to confirm the required mutant genotype. To ensure loss of the I-SceI delivery vector, the mutant strain was grown at 37°C, and loss of the vector confirmed by PCR.

To validate the system and assess its efficiency, we chose to delete an essential region of the undecylprodiginine (Red) biosynthetic gene cluster of *S. coelicolor* that contained the cluster-situated regulatory gene *redD* and the polyketide synthase gene *redX* (Fig. 1^7^;). Red is a red-pigmented tripyrrole compound that remains associated with the mycelium, thus providing a facile visual screen for loss of Red production and a quantitative estimation of deletion frequency.

### First cross-over in one of the homologous regions flanking *redDX*

*Streptomyces* *coelicolor* M1141^8^ is a derivative of *S. coelicolor* M145^9^ in which the gene cluster for production of the blue-pigmented polyketide antibiotic actinorhodin has been deleted. Thus colonies of M1141 are red in appearance, while derivatives that are unable to produce Red would be cream in colour.

pIJ12740, containing sequences flanking *redD* (*trkA*) and *redX* (*redW*) and the adjacent I-SceI recognition site, was introduced into *S. coelicolor* M1141 from *Escherichia coli* by conjugation. Apramycin-resistant exconjugants, all pigmented due to their ability to synthesise Red, were isolated and the presence of the non-replicative plasmid confirmed by PCR. Integration by homologous recombination through *trkA* was confirmed for some of the exconjugants using primers corresponding to the chromosomal sequence to the left of *trkA* (as shown in Fig. 1) and to a sequence internal to *redW* (data not shown). One of these exconjugants was designated M1141Δ*red*_int (for Δ*red* integrant).

### Delivery and expression of I-SceI to select for the second homologous recombination event

**Figure 2:**
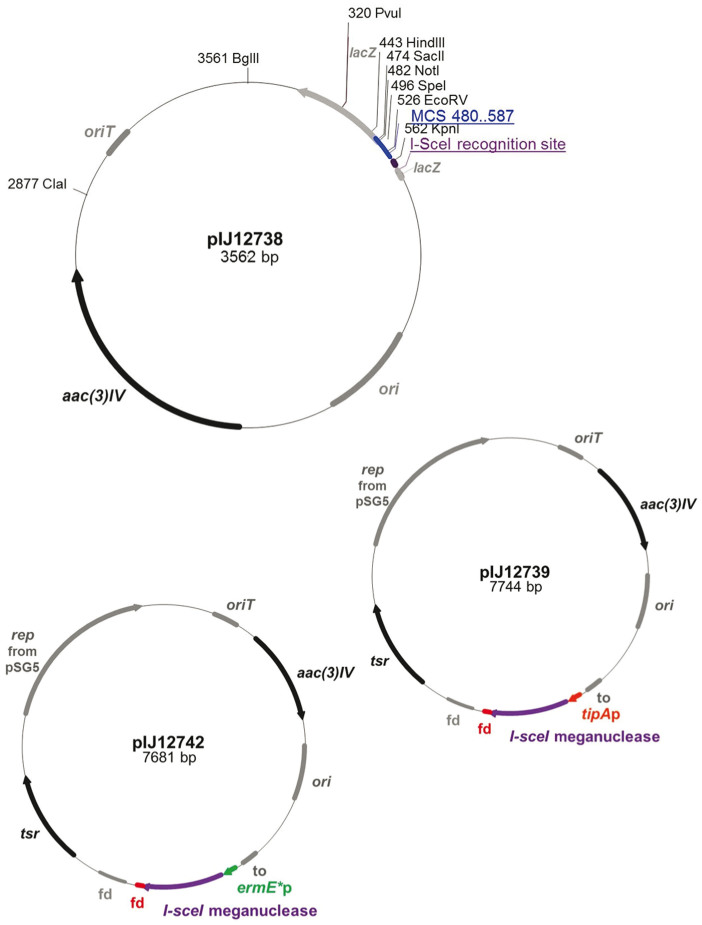
Restriction maps of the plasmids constructed in this study. *aac(3)IV*, apramycin resistance gene; *ori, E. coli* origin of replication; *oriT*, origin of transfer; MCS, multiple cloning site; *lacZ*, α fragment of *lacZ*; *rep*, origin of replication from pSG5; *tsr*, thiostrepton resistance gene; fd, transcriptional terminator from phage fd; to, transcriptional terminator; *tipA*p, thiostrepton inducible *tipA* promoter; ermE*p, mutated constitutive promoter from *ermE*.

**Figure 3:**
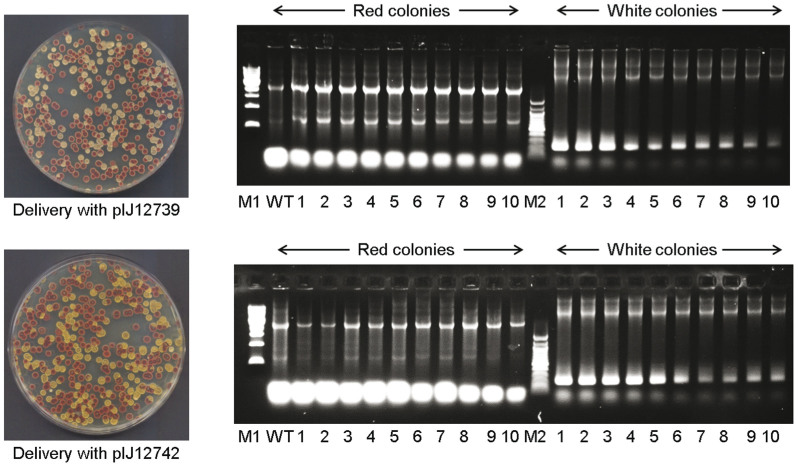
Phenotype and genotype verification of wild type and mutant exconjugants of M1141Δ*red*_int. R5 agar plates containing exconjugants resulting from conjugation of either pIJ12739 (top) or pIJ12742 (bottom). Colony PCR (to the right of each agar plate) carried out on ten red (M1141 with intact *red* gene cluster, expected fragment size 2697 bp), and ten cream (M1141Δ*red*, expected fragment size 160 bp) randomly selected exconjugants confirmed that the phenotypes corresponded to the presence or absence of *redDX*, respectively. M1, 1 kb ladder (New England Biolabs); M2, 100 bp ladder (New England Biolabs). WT, genomic DNA from M1141 used as a control.

To select for second cross-over recombinants, pIJ12739 (Fig. 2), containing the codon optimised I-SceI gene under the control of the thiostrepton inducible *tipA* promoter, was introduced into M1141Δ*red*_int by conjugation from *E. coli* and exconjugants selected by overlaying the agar plate with thiostrepton. Spores were collected directly from the conjugation plate, diluted and plated on R5 agar containing nalidixic acid (to prevent growth of the *E. coli* donor strain) and the number of red and cream colonies on each plate counted (Fig. 3). After examining over 2000 exconjugants from each of four independent matings, the frequency of colonies displaying a Red^−^ phenotype after the second crossover event varied between 29 and 52 percent. Ten cream and ten red colonies were analysed by PCR to confirm deletion of *redDX* in the cream isolates (M1141Δ*red*), reversion to the wild type genotype in the red colonies (Fig. 3), and loss of the pIJ12740 backbone by homologous recombination in all of them (data not shown).

Induction of the *tipA* promoter by thiostrepton requires the presence of the *tipA* gene^10^, which is not present in all actinomycetes. As an alternative strategy, and to overcome this potential limitation, pIJ12742 (Fig. 2), containing the codon optimised I-SceI gene under the control of the strong constitutive ermE* promoter, was delivered to the first cross-over recombinant following exactly the same protocol as described above for pIJ12739. After screening over 2000 colonies from each of three independent conjugations, the frequency of colonies displaying a Red^−^ mutant phenotype varied between 27 and 49 percent, similar to the results obtained with pIJ12739. Again, ten of the cream and ten of the red colonies were analysed by PCR to verify that the phenotype of the colony corresponded to the predicted genotype (Fig. 3) and that the pIJ12740 backbone had been lost by a second homologous recombination event.

### Loss of the temperature-sensitive I-SceI delivery vectors pIJ12739 and pIJ12742

Both pIJ12739 and pIJ12742 are derivatives of pGM1190, an intermediate copy number, conjugative plasmid containing the temperature-sensitive replication origin of pSG5^11^. When exconjugants carrying either pIJ12739 or pIJ12742 are grown above 34°C, the plasmids are unable to replicate and are lost. After plasmid curing, the exconjugants can be grown at their lower optimal temperature. Growth of ten red and ten cream colonies containing each of the I-SceI delivery plasmids on SFM agar at 37°C resulted in complete loss of each of the pGM1190 derivatives, confirmed by their sensitivity to thiostrepton and PCR analysis (data not shown). Thus, after growth at the non-permissive temperature, all of the M1141Δ*red* exconjugants with a marker-less deletion of *redDX* had also lost the nuclease delivery vector, leaving a mutant with no introduced DNA or antibiotic resistance markers.

Interestingly, when *S. coelicolor* M1141Δ*red* derivatives carrying either pIJ12739 or pIJ12742 were passaged through two rounds of sporulation at 30°C in the absence of thiostrepton, 60 percent of the resulting colonies were sensitive to the antibiotic suggesting loss of the plasmids, which was confirmed by PCR analysis (data not shown). This inherent instability in the absence of antibiotic selection could be useful when working with any strains that may not be able to grow at higher temperatures.

## Discussion

In bacteria, double stranded breakage of the chromosome is a lethal event unless the lesion is repaired by homologous recombination. We have taken advantage of the mitochondrial homing endonuclease I-SceI to select for second cross-over recombinants, with 27–52 percent yielding the required mutational event. In this study, we have demonstrated the utility of this approach by constructing a two plasmid system to create a marker-less deletion in *S.* *coelicolor*. This system has also been used to efficiently delete, in an iterative manner, entire gene clusters from the genome of *Streptomyces* *venezuelae* (Neil Holmes, personal communication). In this study we used conjugation from *E. coli* for plasmid transfer (conjugation is the most widely-used procedure for introducing DNA into actinomycetes), but in principle protoplast transformation could be used instead with targeted strains for which conjugation protocols do not yet exist. In addition to being readily applicable to well-studied academic and industrial strains (where there is clear potential for use in the construction of engineered strains with improved expression of heterologous genes and gene clusters), we believe that the system described here will be particularly useful when attempting to make mutants in novel and genetically uncharacterised actinomycetes, especially if they exhibit low levels of homologous recombination and/or fail to sporulate, which generally provides a very useful means of segregating wild type and mutant genomes in a multi-nucleoid mycelial organism. If dealing with a non-sporulating strain, mycelia from each of the conjugation plates could be harvested, fragmented (for example, by using a Potter homogeniser) and then subjected to the same protocol.

## Methods

### Bacterial strains, plasmids and general methods

The strains and plasmids used and generated in this study are listed in Table 1. Plasmid isolation, PCR amplification and *E.* *coli* growth and manipulation were carried out following standard methods^12^,^13^,^14^. *S. coelicolor* strains were grown on SFM and R5 agar as described previously^9^.

### Plasmid construction

Oligonucleotides generated and used in this study are described in Table 2. DNA manipulation and cloning were carried out according to standard protocols^12^. Plasmid constructs were confirmed by sequencing. A fragment containing the *tcp830* promoter^15^, followed by the synthetic I-SceI gene codon optimised for actinomycetes, the *Streptomyces* *lividans* multi-copy plasmid pIJ101 terminator region^16^, a multiple cloning site (MCS) containing the following unique sites: SacII, NotI, XbaI, SpeI, PstI, EcoRI and EcoRV and the I-SceI 18 bp recognition sequence was synthesised by GeneScript (Piscataway, USA) and flanked by HindIII and MfeI restriction sites. The fragment was provided on the plasmid pUC57-Simple_SceI. This plasmid was treated with XbaI and SspI, and the 117 bp fragment containing the MCS followed by the I-SceI recognition site was cloned into pKC1132^5^ cut with XbaI and EcoRV to generate pIJ12738 (Fig. 2).

To construct the plasmid carrying the codon optimised I-SceI gene, pUC57-Simple_SceI was cut with NdeI and EcoRI to generate a 853 bp fragment that was cloned into pGM1190^11^ cleaved with the same enzymes. This generated pIJ12739 (Fig. 2) in which the I-SceI gene is under the control of the inducible *tipA* promoter^17^.

To express the I-SceI gene from the strong constitutive ermE* promoter^18^, a 806 bp NdeI-SacII fragment containing the gene was excised from pIJ12739 and cloned into pIJ12551^19^ digested with the same enzymes to generate pIJ12590. A 1055 bp fragment containing ermE*p-I-SceI gene was amplified by PCR using oligonucleotides containing SnaBII or EcoRI restriction sites (Table 2). This fragment was cloned into pGM1190 digested with the same enzymes to generate pIJ12742 (Fig. 2).

### Construction of deletion mutants

Chromosomal regions flanking the adjacent cluster-situated regulatory gene *redD* and the polyketide synthase gene *redX* of the undecylprodiginine (Red) biosynthetic gene cluster were amplified by PCR and cloned into pIJ12738. Both genes are essential for Red biosynthesis^20^,^21^. The flanking region 5′ *of redD* was amplified to generate a 1516 bp fragment with terminal XbaI and BamHI sites and the region 3′ of *redX* was amplified to generate a 1419 bp fragment with terminal BamHI and KpnI sites. These two flanking fragments were cloned into pIJ12738 digested with XbaI and KpnI to generate pIJ12740 with the I-SceI site adjacent to the introduced *red* gene fragments. pIJ12740 was then introduced into *E. coli* ET12567/pUZ8002^22^ by transformation. Conjugation between *E. coli* ET12567/pUZ8002 carrying the *oriT*-containing plasmid and *Streptomyces* *coelicolor* M1141 was carried out as previously described^9^. Chromosomal integration of pIJ12740 generated M1141Δ*red*_int which was still capable of Red production. pIJ12739 or pIJ12742 carrying the I-SceI gene was then conjugated into M1141Δ*red*_int to induce a double strand break at the introduced I-SceI site. Ten individual red and cream colonies isolated from each of the conjugations were streaked on SFM agar and grown at 37°C until sporulation. Spores were harvested and plated on SFM agar with or without thiostrepton. No colonies grew in the presence of thiostrepton, indicative of plasmid loss, which was confirmed by PCR analysis (data not shown).

## Author Contributions

L.T.F.-M. and M.J.B. conceived and designed the project, and wrote the manuscript; L.T.F.-M. performed the experiments.

## Figures and Tables

**Table 1:** Strains and plasmids used in this study

Strain/Plasmid	Genotype/Description	Reference
*Escherichia coli* DH5α	F^−^ ϕ80 *lacZ*ΔM15 Δ(*lacZYA*-*argF*)U169 *recA*1 *endA*1 *hsdR*17 (r_k_^−^, m_k_^+^) *phoA* *supE*44 *thi*-1 *gyrA*96 *relA*1 λ^−^	Invitrogen™
*E. coli* ET12567	*dam-13::Tn9 dcm-6 hsdM* Cml^R^, carrying helper plasmid pUZ8002	22
*S. coelicolor* M1141	M145 derivative Δ*act*	8
*S. coelicolor* M1141Δ*red*_int	M1141 containing pIJ12740	This study
*S. coelicolor* M1141Δ*red*	M1141 Δ*redDX*	This study
pUC57-Simple_SceI	pUC57-Simple with *tcp830*p-I-SceI gene-*fd* terminator-MCS-I-SceI site	GeneScript (Piscataway, USA)
pKC1132	Conjugative vector, non-replicative in *Streptomyces*, Apr^R^	5
pIJ12738	pKC1132 with MCS and I-SceI site from pUC57-Simple_SceI	This study
pGM1190	*tsr* (Thi^R^), *aac(3)IV* (Apr^R^), *oriT, to* terminator, *tipAp*, RBS, *fd* terminator	11
pIJ12739	pGM1190 with *tipA*p-I-SceI gene, Thi^R^	This study
pIJ12551	Conjugative and ϕC31-integrative vector, Apr^R^	19
pIJ12590	pIJ12551 with I-SceI gene	This study
pIJ12742	pGM1190 with ermE*p-I-SceI gene, Thi^R^	This study
pIJ12740	pIJ12738 with *redXW* left and right flanking sequences	This study

**Table 2:** Oligonucleotides used in this study

Oligonucleotide	Sequence	Notes
ermESceI_F_SnaBI	AATACGTAGGATCCAGCCCGACCCGAGC	Amplify ermE*p-I-SceI gene fusion from pIJ12590
ermESceI_R_EcoRI	ACGAATTCGATATCGCGCGCGG	Amplify ermE*p-I-SceI gene fusion from pIJ12590
redD_left_flank_XbaI_F	AATCTAGAGGACTCGTTGAAGAGCCACT	Amplify the region 5′ to *redDW*
redD_left_flank_BamHI_R	TTGGATCCGTTCTCCGCACTCCCATGA	Amplify the region 5′ to *redDW*
redX_right_flank_BamHI_F	TTGGATCCCACCTGTTGATCGAGGAAGG	Amplify the region 3′ to *redDW*
redX_right_flank_KpnI_R	ATAGGTACCCACCGAGATGCTCTCGAAGT	Amplify the region 3′ to *redDW*
KC1132_flank_SacII_check_F	GTTTTCCCAGTCACGACGTT	Confirm presence of pIJ12740 in M1141
KC1132_flank_EcoRV_check_R	TGTGGAATTGTGAGCGGATA	Confirm presence of pIJ12740 in M1141
Red_deletion_check_F	CTGTACAACTTCGGGGGATG	Confirm *redXW* deletion in exconjugants
Red_deletion_check_R	GCGTCGAAGTCGAAGTTCAT	Confirm *redXW* deletion in exconjugants
SceI_check_F	CATGCAGTTCGAGTGGAAGA	Confirm presence of pIJ12739 or pIJ12742
SceI_check_R	CGGGATGGTCTTCTTGTTGT	Confirm presence of pIJ12739 or pIJ12742
